# Mid-term Results Following Reverse Shoulder Arthroplasty and the Role of Navigation in the Management of Glenoid Bone Loss

**DOI:** 10.7759/cureus.54633

**Published:** 2024-02-21

**Authors:** Georgios Saraglis, Hamdip Singh, Zain Charfare, Gbemisola Jones Olujinmi, Gertrud Devecseri, Adeniyi Agbaje, Joby George Malal

**Affiliations:** 1 Orthopaedics and Trauma, Bedfordshire Hospitals NHS Foundation Trust, Bedford, GBR

**Keywords:** orthopedic navigation, glenoid bone defect, surgical navigation system, reverse total shoulder replacement, reverse shoulder arthoplasty, navigation system

## Abstract

Background

Inaccurate positioning of the glenoid component has been well described as the most common cause of early failure following a reverse shoulder arthroplasty (RSA). Among the latest developments in operative technique, three-dimensional preoperative planning and navigation intraoperative systems have been developed to improve the accuracy of the baseplate positioning during RSA. The primary purpose of this retrospective analysis was to investigate the mid-term results of patients who underwent an elective RSA or for acute highly comminuted proximal humerus fractures. The secondary goal was to investigate the role of navigation in the execution of preoperative planning, especially in the management of glenoid bone loss.

Methodology

In total, 101 cases were included in this study. Patients were divided into the following two groups: 88 cases of RSA performed without the use of navigation (conventional RSA) and 13 cases performed using intraoperative navigation (navigated RSA). For all patients included in the study, preoperative planning software was employed. Patient demographics, gender, past medical history, indication of procedure, operated site, type of glenoid component used, length of baseplate screws, and clinical assessment scores (Oxford Shoulder Score, OSS) were reported for all patients. Cases of revision shoulder arthroplasty were excluded from this study.

Results

The postoperative clinical assessment of patients revealed that following RSA, all patients improved significantly with a consistently upward trend of the OSS noted for both groups (conventional and navigated RSA) throughout the postoperative assessment. Despite no statistically significant difference detected, the clinical scores of the navigated RSA group outperformed those of the conventional RSA group in the postoperative period. A higher incidence of augmented baseplate use was noted in the navigated RSA group than in the conventional group (23.07% vs. 5.68%, p < 0.001).

Conclusions

Our results indicate that the use of intraoperative navigation appears to be a valuable tool in preoperative planning, providing accurate positioning of the baseplate, a better understanding of the glenoid anatomy, and real-time monitoring of the length and direction of the baseplate screws. It is difficult to conclude if the use of navigation leads to superior clinical outcomes, and the cost-effectiveness of its use needs to be further analyzed. Prospective randomized trials are required to assess the cost-effectiveness of routine use of navigation in RSA.

## Introduction

The indications for reverse shoulder arthroplasty (RSA) have expanded widely, including for patients with irreparable rotator cuff tears, severe rheumatoid arthritis, complex proximal humerus fractures, recurrent chronic shoulder dislocations, rotator cuff arthropathy, and revision procedures [1,2]. Numerous studies focusing on the clinical outcomes of RSA have been reported, focusing on the short-term clinical outcomes following RSA for proximal humerus or elective procedures [3,4] with a small sample size of approximately 30 patients. As the number of RSA cases rises rapidly, the benefits in terms of functional outcomes are becoming clearer along with the need for understanding the most common complications [5].

Among the most well-described complications of RSA, the failure of the glenoid component due to instability or loosening is one of the most common complications after RSA leading to revision surgery [6]. The poor position of the baseplate, the inaccurate inclination, and the overall alignment of the glenoid prosthesis are strongly linked with early glenoid failure and the need for revision surgery [7,8]. As indicated in previous studies [9,10], the micromotion and stress concentration that develops following inaccurate version and orientation of the baseplate leads to bone resorption and loosening, whereas the excessive reaming of the glenoid inevitably removes valuable subchondral bone which potentially causes subsidence of the glenoid component [9,10].

To overcome the difficulties in correct glenoid preparation and more accurate glenoid component positioning, computer-assisted technology, three-dimensional (3D) preoperative surgical planning software, and patient-specific instruments have been used [11,12]. The advantages of navigation systems include more accurate screw direction and baseplate alignment, intraoperative assessment of glenoid orientation (version and inclination), real-time visualization of screw direction, and, when combined with preoperative 3D planning software, more accurate placement of the glenoid component, especially in cases with pre-existing glenoid bone loss [13,14].

The objective of this study was to investigate the mid-term results of patients of all age groups who underwent a primary reverse polarity shoulder replacement at a single orthopedic unit, under one surgeon, in the United Kingdom. This study aimed to assess the mid-term results between patients who underwent an elective procedure for irreparable rotator cuff deficiencies, rotator cuff arthropathy, rheumatoid arthritis, or recurrent chronic shoulder dislocations and those with acute complex proximal humerus fractures. Secondary objectives were to assess if the use of a navigation system led to superior clinical outcomes and investigate the role of navigation in cases of glenoid bone loss.

## Materials and methods

This was a retrospective analysis of 101 patients who underwent a reverse polarity shoulder replacement between 2015 and 2021 under the same surgeon using the same implant (Equinoxe system, Exactech, Gainesville, FL, USA). Of the 101 cases of RSA included in the study, 13 were performed using intraoperative navigation (GPS navigation system, Exactech, USA) (navigated RSA). The remaining 88 cases were performed using the conventional method without the use of intraoperative navigation (conventional RSA). For all cases included in the study, preoperative planning was performed using preoperative software (Equinoxe Planning Software App, Exactech Blue Otho, France).

Patient demographics, gender, operative side, glenoid component option, screw length for the baseplate fixation, and indication for the surgical procedure were recorded for all patients. Preoperative and postoperative clinical assessment of all patients was performed using the 48-point Oxford Shoulder Score (OSS), whereas the preoperative glenoid morphology was assessed using 3D computed tomography (CT), and was subdivided into different types according to Walch classification (in axial plane) [14].

Preoperative planning (Exactech Equinoxe system)

All patients included in the study underwent an RSA using the Equinoxe Exactech system (Exactech, Gainesville, FL, USA) which is based on the principle of a medialized glenoid and lateralized humerus [15]. During preoperative planning, once glenoid erosion is identified, an augmented glenoid baseplate is used to correctly adjust the correct version and inclination of the glenoid. In our study, glenoid implant options included standard and 8° posterior augmented baseplate. The glenosphere options included standard glenosphere and +4 mm (more lateralized) glenosphere.

**Figure 1:**
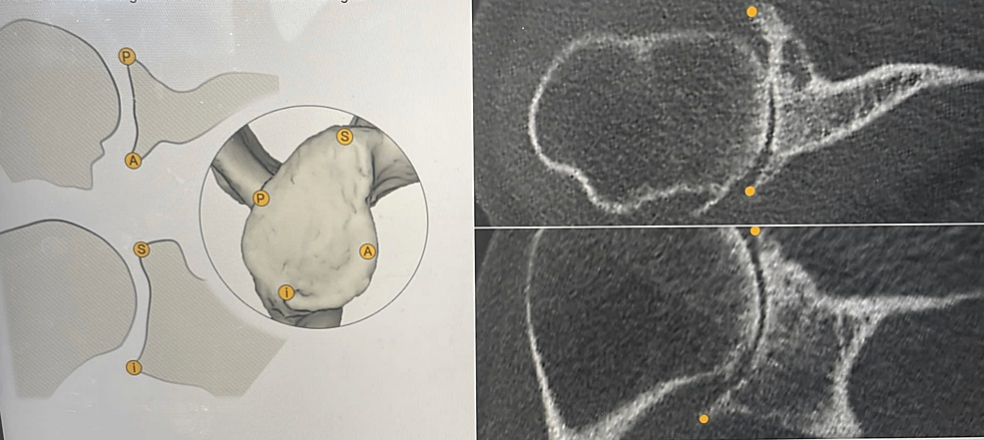
Preoperative planning software (Equinoxe planning software, Exactech BlueOrtho): three-dimensional analysis of the glenoid morphology by assessing the glenoid margins. A: Anterior border of the glenoid. P: Posterior border of the glenoid. S: Superior margin of the glenoid, I: Inferior margin of the glenoid. Yellow circles: Anterior-posterior and superior-inferior margins of the glenoid (axial and coronal planes of computed tomography).

**Figure 2:**
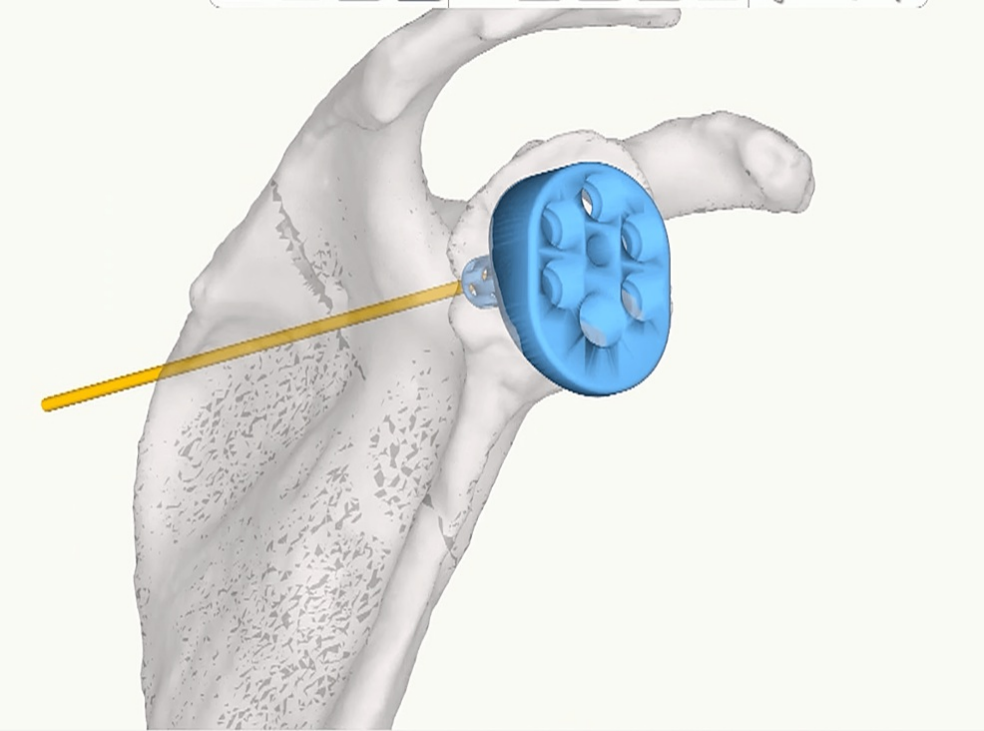
Preoperative planning software assessing the position of the baseplate and orientation along Friedman’s axis (yellow line). Yellow line: Friedman’s axis passing through the center of the glenoid baseplate (blue).

Before the preoperative planning, CT was acquired in the axial plane, and after the segmentation of the scapula, Friedman’s axis was calculated from the center of the glenoid to the medial border of the scapula [16]. During the application of the planning software, different virtual glenoid baseplates can be tested to match the native anatomy of the patient’s glenoid, and the position of the baseplate is determined along Friedman’s axis at 0° of version and 0° of inclination (Figures 1, 2).

In cases where the preoperative software reveals excessive glenoid bone loss, an augmented glenoid baseplate can be used to minimize the excessive intraoperative reaming. When the surgeon completes the surgical planning, this is saved and uploaded to the intraoperative navigation system. In the cases performed without an intraoperative navigation system, the preoperative planning was saved and uploaded in the electronic patient notes for preoperative guidance (conventional RSA).

Surgical technique

For all cases, a standard deltopectoral approach was used. Following the safe positioning of the patient in a beach chair position and after standard preparation and draping, the deltopectoral approach was performed, and following humeral head resection, the glenoid was exposed.

For the conventional RSA, the center of the glenoid was identified by calculating the intersection of the vertical and perpendicular midlines of the glenoid. A starting 2.0 mm Kirschner wire was introduced and reaming of the glenoid was performed in a neutral version and inclination under direct vision. The subsequent central peg hole creation, baseplate insertion, impaction, and screw insertion were performed manually under direct vision.

For the navigated RSA, the coracoid tracker was positioned on the coracoid and several glenoid and coracoid points were registered using the pointer and uploaded to the navigation system. For the navigated RSA and in comparison to the conventional RSA, the glenoid reaming and drilling of the central peg hole were performed according to the preoperative planning and as guided by the navigation system intraoperatively. The baseplate was then inserted, and adequate positioning in line with the preoperative planning was confirmed. Out of the six variable angle screw holes of the baseplate, four screws were used for all cases (navigated and conventional groups).

In the navigated RSA group, the intraoperative navigation system offered a screen display of the trajectory of the drilling in real time. After the insertion of the baseplate screws, locking caps were positioned on the screw heads before inserting the glenosphere. Following the preparation of the glenoid, standard humeral preparation was performed and the humeral implants were inserted.

Statistical analysis

Patient demographics including self-reported gender, operated site, clinical assessment score (OSS), past medical history (diabetes, neurological disorder, rheumatoid arthritis, etc.), the indication of the procedure (trauma vs. elective), type of glenoid implant used (standard baseplate, 8° posterior augmented baseplate, standard glenosphere, +4 mm glenosphere), hospitalization period until discharge, and the length the baseplate screws used were identified for all cases. To identify differences between the conventional and navigated RSA groups, the unpaired t-test was utilized to compare the mean preoperative and postoperative OSS between the two groups, as well as for the comparison of the mean screw length of the baseplate fixation and the average mean stay in hospital until medical discharge. A p-value of less than 0.05 was considered statistically significant.

## Results

There was no statistically significant difference in patients’ demographics between the two groups (Table 1). Four postoperative complications were noted in the conventional RSA group: one case of postoperative dislocation in the fifth week postoperatively, leading to revision surgery using a constraint polyethylene; one case of deep infection requiring staged revision surgery; one case of superficial suture granuloma, recovering completely following administration of oral antibiotics by the local general practitioner (GP); and one case of postoperative medial cutaneous nerve of the forearm palsy, which fully recovered with conservative management at eight weeks postoperatively. In the navigated RSA group, one case of intraoperative undisplaced coracoid fracture was noted, which was treated conservatively (Table 1).

**Table 1:** Patient demographics. RSA: reverse shoulder arthroplasty; N: number of cases

	Conventional RSA	Navigated RSA	P-value
Number of patients (N)	88	13	
Gender: male/female	41/47	4/9	0.412
Operative side: right/left	52/36	3/10	0.65
Indication of procedure: trauma/rotator cuff arthropathy	23/65	0/13	
History of diabetes (number of cases)	20	2	
History of neurological disorders (Parkinson’s disease, etc.)	16	2	
History of rheumatoid arthritis	6	1	
Mean stay until medical discharge (in days)	1.32 days (1–5 days)	1.28 days (1–5 days)	0.90
Postoperative complications			0.42
Dislocations	One case of postoperative dislocation requiring revision to constraint polyethylene	-	
Infections	One case of deep infection requiring a staged revision procedure. One case of superficial suture granuloma, which resolved	-	
Nerve palsy/fractures	One case of medial cutaneous nerve of the forearm palsy, which completely resolved	One case of coracoid fracture	

Clinical scores

The mean preoperative and postoperative OSSs were determined for all patients of both groups. For all patients included in the study, clinical assessment was performed at six weeks, three months, six months, and annually postoperatively (up to six years postoperatively). The statistical analysis of the mean OSS between the two groups did not reveal any statistically significant difference between the two groups. In both conventional and navigated RSA, the OSS followed an upward trend, but a consistently better clinical performance (as indicated by the OSS) was noted for the navigated RSA group throughout the follow-up period (Table 2).

**Table 2:** Oxford Shoulder Score assessment. RSA: reverse shoulder arthroplasty; OSS: Oxford Shoulder Score

	Conventional RSA	Navigated RSA	P-value
Preoperative mean OSS	16.31	17.46	0.68
Six-week mean postoperative OSS	22.14	22.25	0.97
Three-month mean postoperative OSS	26.84	27.72	0.81
Six-month mean postoperative OSS	34.61	35.42	0.82
One-year mean postoperative OSS	37.10	41.06	0.3
Two-year mean postoperative OSS	37.88	42.11	0.21
Three-year mean postoperative OSS	39.31	-	-
Four-year mean postoperative OSS	40.23	-	-
Five-year mean postoperative OSS	41.122	-	-
Six-year mean postoperative OSS	44.38	-	-

Use of augmented baseplates and augmented glenospheres

In the conventional RSA group, an augmented baseplate was used in five cases (5.68% of all conventional cases) and an augmented glenosphere in three cases. All augmented baseplates included 8° posterior augmented baseplates and all augmented glenospheres were +4 mm lateralized glenospheres. For the navigated RSA group, three cases (23.07%, p < 0.001) of augmented baseplate were identified (all cases were 8° posterior augmented baseplate), and no cases of augmented glenosphere were recorded.

Screw length of the baseplate fixation

For all cases in both groups, four screws were used for the baseplate fixation (superior, inferior, posterior-inferior, and anterior-inferior). The length of the screws used was collected for all cases. The mean screw length of the navigated RSA group was longer than the mean screw length of the conventional RSA group (27.88 mm vs. 25.01 mm). Due to the heterogeneity between the two groups (88 vs. 13 cases), it was difficult to conclude if the above difference was statistically significant (Table 3).

**Table 3:** Mean glenoid screw length and glenoid type (Walch classification). RSA: reverse shoulder arthroplasty; SD: standard deviation; N: number of cases

	Conventional RSA	Navigated RSA
Overall screw length, mm, mean (SD)	25.01(4.4)	27.88(4.2)
Glenoid type A1/A2, N	44/15	2/3
Glenoid type B1/B2/B3, N	17/8/4	4/3/0
Glenoid type C, N	0	1
Glenoid type D, N	0	0

## Discussion

The purpose of this study was to focus on the mid-term results following an RSA and the implications of the intraoperative navigation system for the accurate positioning of the baseplate component, especially in cases with significant bone loss.

Among the most well-described prognostic factors predisposing to early failure of the reverse shoulder replacement is the poor positioning of the glenoid component [17]. Positioning the glenoid component in excessive superior inclination can lead to detrimental effects such as scapular notching, medial polyethylene wear, scapular impingement, and, as a result, early aseptic loosening of the implant [18,19]. In contrast, an excessive inferior inclination of the glenoid component of more than 10° can lead to increased shear force concentration on the baseplate and catastrophic failure [20].

Excessive retroversion of the glenoid component can also lead to early glenoid failure. More than 10° of glenoid retroversion has been associated with increased stress concentration on the glenoid baseplate and increased posterior glenoid bone absorption [21], whereas retroversion above 15° has been linked to increased incidence of osteolysis around the glenoid center peg [22].

To avoid such common and catastrophic complications, 3D planning software, intraoperative navigation systems, and computer-assisted surgical software have been developed to achieve accurate baseplate orientation. The use of preoperative planning software has increased rapidly over the last decade, offering a more accurate understanding of the glenoid anatomy and orientation leading to an accurate selection of the appropriate glenoid component [12]. In cases of glenoid bone loss, before the introduction of baseplate augments, excessive reaming of the glenoid was performed to achieve adequate correction of the glenoid deformity; however, as a result, the center of rotation was excessively medialized leading to poor baseplate fixation, reducing the arc of motion and instability [23].

These limitations of excessive reaming of the glenoid can be avoided by the use of augmented baseplates. The augmented baseplate reduces the need for glenoid bone removal and maximizes the correction of the glenoid deformity [24]. In the study of Kida et al. [15], the use of preoperative planning was linked to a higher incidence of augmented baseplates being used in comparison to cases that did not receive preoperative planning. This could be possibly explained by the fact that preoperative software allows for a better understanding of the glenoid morphology; however, augmented baseplates can potentially lead to lateralization of the glenoid and decreased postoperative range of movement [25].

The use of augmented baseplates has increased after the introduction of navigation systems [24]. Intraoperative navigation systems use the data retrieved from preoperative surgical planning to determine the accurate placement of the baseplate. In the study of Kida et al. [15], a higher precision of baseplate alignment was noted in navigated RSA than in conventional RSA, with similar findings reported by other studies [26,27].

Another superiority of the intraoperative use of navigation is the real-time intraoperative monitoring of the direction of the screw insertion of the baseplate. The primary stability of the baseplate has been associated with the length of the screw used. Longer baseplate screws lead to improved glenoid stability [28,29]. In our study, the average screw length in the navigated RSA was found to be longer than the conventional RSA group (Table 3). Apart from a longer screw length, the navigation system appears to provide a safe zone for the insertion of the baseplate screws. Malposition of the baseplate screws has been associated with perforation of the glenoid vault and in some cases acromion fractures by the perforation of the upper screw [29].

There were some limitations of this study. This was a retrospective non-randomized cohort study including patients who underwent an elective procedure as well as patients who underwent an RSA for acute highly comminuted proximal humerus fractures. Additionally, the number of navigated RSA was limited (13 cases vs, 88 cases of conventional RSA) and with a shorter follow-up in comparison to the conventional RSA group. This is mainly because the intraoperative navigation system has been available in our institution since 2017; hence, the number of navigated RSA cases included in our study was relatively limited in comparison to the conventional RSA group. Furthermore, as there were few cases of severe glenoid bone loss in both groups, the number of augmented baseplates used was relatively low.

Despite these limitations, to our knowledge, this study is among the few studies including an adequate number of cases (>100 cases) with a long-term follow-up with all cases performed under one surgeon, focusing on the implications and the role of navigation in the management of glenoid bone loss. Despite no statistically significant difference in the clinical outcomes between the two groups, in our study, the use of navigated RSA was linked with a higher incidence of augmented baseplate use, which can potentially provide a more stable glenoid baseplate fixation with fewer complications in cases with underlying glenoid bone loss.

## Conclusions

This was a retrospective cohort study presenting the mid-term results following an RSA, focusing on the implications of preoperative planning and the role of navigation in the management of glenoid bone loss. Both the conventional and navigated RSA groups seem to have satisfactory clinical outcomes with no statistically significant difference detected between the groups.

The use of navigation was linked with a higher incidence of augmented baseplate use, which can potentially lead to superior and more stable glenoid component fixation in cases of glenoid bone loss. Furthermore, the navigation system along with preoperative software enables a more accurate and precise understanding of the glenoid morphology, along with real-time monitoring of the direction of the glenoid screws, reducing the incidence of intraoperative complications.

As the navigation system adds an extra cost to the national health system, it is difficult to draw definite conclusions as to whether its use is cost-effective. Further studies, including prospective randomized trials, are necessary to assess component survivorship and the implications of the navigation in RSA.
